# Editorial: Cell Stress, Metabolic Reprogramming, and Cancer

**DOI:** 10.3389/fonc.2018.00236

**Published:** 2018-06-25

**Authors:** Sergio Giannattasio, Mario G. Mirisola, Cristina Mazzoni

**Affiliations:** ^1^Institute of Biomembranes, Bioenergetics and Molecular Biotechnologies, National Research Council of Italy, Bari, Italy; ^2^Department of Surgical, Oncological and Oral Sciences, Section of Medical Oncology, University of Palermo, Palermo, Italy; ^3^Department of Biology and Biotechnologies “C. Darwin”, Pasteur Institute-Cenci Bolognetti Foundation, Sapienza University of Rome, Rome, Italy

**Keywords:** cancer, mitochondria, hypoxia-inducible factor 1 alpha, ataxia-telangiectasia mutated, l-lactate, glutamine, antioxidant response, epithelial-to-mesenchymal transition

**Editorial on the Research Topic**

**Cell Stress, Metabolic Reprogramming, and Cancer**

The hallmarks of cancer comprise six biological capabilities acquired during the multistep development of human tumors: sustaining proliferative signaling, evading growth suppressors, resisting cell death, enabling replicative immortality, inducing angiogenesis, and activating invasion and metastasis (1). Mitochondria, beyond being the site of aerobic respiration, are at the crossroads of a variety of metabolic and signaling pathways resulting key regulatory organelles in cell life and death decision. Thus, it is no surprise that genomic, functional, and structural mitochondrial alterations have been associated with cancer and that mitochondria have become a pharmacological target in cancer therapy (2). Proliferating tumor cells show increased glycolysis and convert the majority of glucose to l-lactate, even in normoxic conditions. This is known as the Warburg effect. Actually, in many tumors, mitochondria are not defective in oxidative phosphorylation, and in the last decade, the molecular basis of Warburg effect has been reconsidered in the context of a set of concerted changes in energy metabolism and mitochondrial function that support tumorigenesis. This process, referred to as reprogramming of energy metabolism, is an emerging hallmark of cancer development (3, 4). This Research Topic presents one review, five mini-reviews, and an opinion article on the achievements and perspectives of studies on important aspects of cancer cell metabolic reprogramming whose mechanisms and regulation are still largely elusive. It also sheds light on certain novel functional components, which rewires cell metabolism in tumor transformation.

Metabolic reprogramming is driven by oncogenic changes of specific cell-signaling pathways and tumor microenvironment (5). The Mini-Reviews by Iommarini et al. (6) and Dahl and Aird (7) highlight what is currently known about the non-canonical function and regulation of hypoxia-inducible factor 1 alpha (HIF-1α) and ataxia-telangiectasia mutated (ATM) protein kinase, respectively. Iommarini et al. (6) review and discuss the non-canonical regulation of HIF-1α expression and stabilization in cancer cells, focusing on factors, which cause pseudohypoxia (HIF-1α stabilization in normoxic conditions) or fail to stabilize HIF-1α in low oxygen atmosphere (pseudonormoxia). The ATM protein kinase has been extensively studied for its role in the DNA damage response and its association with the disease ataxia telangiectasia. Dahl and Aird’s review (7) highlights our current knowledge about ATM’s regulation of carbon metabolism, the implication of these pathways in cancer, and the development of ATM inhibitors as therapeutic strategies for cancer.

It is well established that glucose is uniquely capable of supporting Warburg metabolism (or aerobic glycolysis), in which pyruvate is converted to lactate through a process that is coupled to ATP production in the cytoplasm. Such metabolic reprogramming and nutrient sensing is an elaborate way by which cancer cells respond to high bioenergetic and anabolic demands during tumorigenesis. Ždralević et al. (8) in their Mini-Review discuss the benefits and limitations of disrupting fermentative glycolysis at different levels of the pathway in order to find the most effective mode to overcome cancer cell metabolic plasticity that seriously limits the use of glycolysis inhibition for impeding tumor growth. With this respect, in view of the existence of a mitochondrial l-lactate dehydrogenase (m-l-LDH), Passarella and Shurr (9) propose in their Opinion a revision of the Cori cycle in all types of cells where mitochondrial metabolism of l-lactate is active.

Beyond the shift of glucose metabolism to aerobic glycolysis, some cancer cells are considered “glutamine addicted” because their growth and proliferation rates depend on the availability of this amino acid. This, together with the role of amino acid metabolism in tumorigenesis, is one of the key aspects of cancer cell metabolism, which is still matter of intense investigations. The Review by Vučetić et al. (10) provides the first unified review on the amino acid dependency of cancer antioxidant defense, a topic that has received more attention recently. Furthermore, the Mini-Review by Scalise et al. (11) provides a deep insight into glutamine transport and mitochondrial metabolism in cancer cell growth, highlighting glutamine transporters of plasma membrane, the key enzyme glutaminase, and other proteins involved glutamine metabolism as novel targets for anti-cancer drug development.

Beyond the metabolic shift toward glycolysis, typical of cancer cells, several evidences have shown that mitochondrial dysfunction provides survival advantage to cancer cells, suggesting that mitochondria have a tumor suppressor function (5). Mitochondrial dysfunction has been implicated in cancer chemoresistance (12). The association between mitochondrial dysfunction and progression to a metastatic phenotype is gradually emerging. Epithelial-to-mesenchymal transition (EMT) allows epithelial cancer cells to assume mesenchymal features, endowing them with enhanced motility and invasiveness, thus enabling cancer dissemination and metastatic spread. The Mini-Review by Guerra et al. (13) in this Research Topic gives an overview on the mechanistic link between EMT and mitochondrial dysfunction fostering the identification of the molecular determinants of the mitochondria-nucleus communication network linking mitochondrial dysfunction with EMT activation, which may provide useful therapeutic targets for treatment and prevention of metastatic cancer.

The contributions to this Research Topic deal with investigations at the leading edge of cancer research and provide an overview on key cellular processes and components, which are the basis of metabolic reprogramming of cancer cells. Inflammation has also been recognized as a hallmark of cancer and is known to play an essential role in the development and progression of most cancers, even those without obvious signs of inflammation and infection (14). Warburg metabolism is a hallmark of immune cells that have the potential to cause inflammation. Recently, Kornberg et al. gave proof of concept that aerobic glycolysis is a therapeutic target for regulating inflammation (15), further confirming the possibility that targeting key enzymes within metabolic pathways will provide new therapeutic options for cancer.

This Research Topic brings witness that research on metabolic reprogramming of cancer cells is coming of age and will still bring with it exciting results to lay the bases for the development of new therapies and the implementation of nutritional regimen for a healthy life as well as the improvement of anti-cancer therapies.

## Author Contributions

SG wrote the first draft of the manuscript; SG, MGM and CM contributed to manuscript revision.

## Conflict of Interest Statement

The authors declare that the research was conducted in the absence of any commercial or financial relationships that could be construed as a potential conflict of interest.

## References

[1] Hanahan D Weinberg RA Hallmarks of cancer: the next generation. Cell (2012) 144:646–74. 10.1016/j.cell.2011.02.013 21376230

[2] Picard M Wallace DC Burelle Y. The rise of mitochondria in medicine. Mitochondrion (2016) 30:105–16. 10.1016/j.mito.2016.07.003 27423788 PMC5023480

[3] Pavlova NN Thompson CB. The emerging hallmarks of cancer metabolism. Cell Metab (2016) 23:27–47. 10.1016/j.cmet.2015.12.006 26771115 PMC4715268

[4] Vander Heiden MG DeBerardinis RJ. Understanding the intersections between metabolism and cancer biology. Cell (2017) 168:657–69. 10.1016/j.cell.2016.12.039 28187287 PMC5329766

[5] Vyas S Zaganjor E Haigis MC. Mitochondria and cancer. Cell (2016) 166:555–66. 10.1016/j.cell.2016.07.002 27471965 PMC5036969

[6] Iommarini L Porcelli AM Gasparre G Kurelac I Non-canonical mechanisms regulating hypoxia-inducible factor 1 alpha in cancer. Front Oncol (2017) 7:286 10.3389/fonc.2017.00286 29230384 PMC5711814

[7] Dahl ES Aird KM Ataxia-Telangiectasia mutated modulation of carbon metabolism in cancer. Front Oncol (2017) 7:291 10.3389/fonc.2017.00291 29238697 PMC5712564

[8] Ždralević M Marchiq I Cunha de Padua MM Parks SK Pouysségur J Metabolic plasiticy in cancers—distinct role of glycolytic enzymes GPI, LDHs or membrane transporters MCTs. Front Oncol (2017) 7:313 10.3389/fonc.2017.00313 29326883 PMC5742324

[9] Passarella S Schurr A l-Lactate transport and metabolism in mitochondria of Hep G2 cells—The Cori cycle revisited. Front Oncol (2018) 8:120 10.3389/fonc.2018.00120 29740537 PMC5924780

[10] Vučetić M Cormerais Y Parks SK Pouysségur J The central role of amino acids in cancer redox homeostasis: vulnerability points of the cancer redox code. Front Oncol (2017) 7:319 10.3389/fonc.2017.00319 29312889 PMC5742588

[11] Scalise M Pochini L Galluccio M Console L Indiveri C Glutamine transport and mitochondrial metabolism in cancer cell growth. Front Oncol (2017) 7:306 10.3389/fonc.2017.00306 29376023 PMC5770653

[12] Guerra F Arbini AA Moro L. Mitochondria and cancer chemoresistance. Biochim Biophys Acta (2017) 1858:686–99. 10.1016/j.bbabio.2017.01.012 28161329

[13] Guerra F Guaragnella N Arbini AA Bucci C Giannattasio S Moro L Mitochondrial dysfunction: a novel potential driver of epithelial-to-mesenchymal transition in cancer. Front Oncol (2017) 7:295 10.3389/fonc.2017.00295 29250487 PMC5716985

[14] Taniguchi K Karin M NF-kappaB, inflammation, immunity and cancer: coming of age. Nat Rev Immunol (2018) 18:309–24. 10.1038/nri.2017.142 29379212

[15] Kornberg MD Bhargava P Kim PM Putluri V Snowman AM Putluri N Dimethyl fumarate targets GAPDH and aerobic glycolysis to modulate immunity. Science (2018) 360:449–53. 10.1126/science.aan4665 29599194 PMC5924419

